# Understanding why the COVID‐19 pandemic‐related lockdown increases mental health difficulties in vulnerable young children

**DOI:** 10.1111/jcv2.12005

**Published:** 2021-04-07

**Authors:** Dolapo Adegboye, Ffion Williams, Stephan Collishaw, Katherine Shelton, Kate Langley, Christopher Hobson, Daniel Burley, Stephanie van Goozen

**Affiliations:** ^1^ Department of Psychology Cardiff University Cardiff South Glamorgan UK; ^2^ Department of Psychological Medicine and Neurology Cardiff University School of Medicine Cardiff South Glamorgan Wales

**Keywords:** anxiety, child mental health, COVID‐19, parent mental health, poverty

## Abstract

**Background:**

The mental health consequences of school closure, social isolation, increased financial and emotional stress, and greater exposure to family conflicts are likely to be pronounced for primary school children who are known to be vulnerable. Data from prior to the pandemic are needed to provide robust assessments of the impact of COVID‐19 on vulnerable children.

**Method:**

The present study capitalises on an ongoing study of primary school children (4–8 years) identified as ‘at‐risk’ for mental health problems by teachers. We collected mental health and socio‐economic data prior to the pandemic and re‐assessed this cohort (*n* = 142) via researcher‐led video calls during the pandemic to evaluate the social and emotional impacts of COVID‐19 for these families.

**Results:**

Mental health problems, particularly anxiety, increased significantly in these children. Parental mental health difficulties (anxiety and depression) were also prevalent. There were higher reports of financial stress during lockdown amongst low‐income families previously identified as living in poverty, prior to the COVID‐19 pandemic. Financial strain was found to indirectly predict increases in child mental health problems through parental mental health.

**Conclusion:**

These findings show that the pandemic exacerbated mental health problems in already vulnerable children. These negative outcomes were explained by financial stress (e.g., lost employment, loss of income and inability to pay bills), which was negatively linked to parental mental health.

## INTRODUCTION

There is an urgent need to assess and understand the psychological and social impacts of the COVID‐19 pandemic and associated lockdown on primary school children, especially those who are already known to be at risk of significant emotional and behavioural problems (Jefsen et al., 2020; Racine et al., 2020). There is also a lack of knowledge about how best to support high‐risk families during and after lockdown(s). It is well known that even in very high‐risk groups there is substantial heterogeneity in the way that individuals cope with stress and trauma; this applies to children at high familial risk of developing mental health problems and to children impacted by other health pandemics (Collishaw, Hammerton, et al., 2016; Collishaw, Gardner, et al., 2016). Some children fare much better than expected. The reasons for this involve multiple protective factors involving child, family and community, and many of these factors are likely to be modifiable, examples being children's coping skills, measures to alleviate poverty and food insecurity (Ridley et al., 2020), and broader community factors, including educational support and engagement from schools (Collishaw, Hammerton, et al., 2016; Collishaw, Gardner, et al., 2016).

**Key points**

- Pre‐pandemic data, as well as data from during the pandemic, are needed to robustly assess the social and emotional impacts of COVID‐19 on vulnerable children and identify how negative consequences can be mitigated. In the current study, we made use of data from both periods.
- During the pandemic, we conducted video calls, using validated interviews and psychometric tools, with 142 primary‐school‐aged children with emerging mental health problems and one parent.
- The lockdown resulting from COVID‐19 had a significantly negative impact on child mental health. Parental anxiety and depression problems were also prevalent during lockdown.
- Financial strain was significantly associated with parental mental health problems, which in turn were significantly associated with child mental health problems.
- Families whose financial circumstances have been seriously adversely affected by the pandemic are in need of financial support, which should benefit children's mental health.
- Interventions to support parental mental health should also help to protect children's mental health.

In the context of COVID‐19, a range of advice for parents already exists,^1^ but what is needed now is robust assessment of the child and family‐related factors that are most strongly associated with better or worse outcomes. Many children and families have seen changes in their everyday lives, including changes in education provision, employment, physical activity and social contact. With the pandemic impacting families financially, and with many parents having to balance work commitments with managing their children at home (also affecting daily routines, such as eating and sleeping) while still carrying out their child's schooling commitments, it is clear that families have been exposed to significant psychological and social stress that may well have affected family functioning (Giallo et al., 2014). Understanding the immediate psychological and social consequences for children, especially those already at risk of significant emotional and behavioural problems, and their families, is essential for rapid development of policies and interventions to mitigate the mental health problems and provide tailored support for vulnerable groups of children during and after the pandemic.

Understanding how COVID‐19 and the associated lockdown have affected children and their families requires comprehensive baseline data that predate the pandemic and can be used to identify modifiable factors that are associated with more resilient outcomes (Holmes et al., 2020). This is even more crucial for children with emerging mental health problems and families for whom there is additional legitimate concern regarding financial stress resulting from the lockdown^2^ and likely disconnection from societal protective structures (including school and health/social care services), as well as the possibility of being affected by increased exposure to domestic violence, parental mental health problems and change of social networks. Our first objective was to generate a detailed understanding of the specific mental health needs of children with emerging mental health problems and the economic profiles of their families, including whether and how these had changed as a result of the COVID‐19 pandemic. Our second objective was to identify modifiable child and family‐related factors (e.g., parental mental health and financial resources) that contribute to risk and resilience and could be targeted for intervention. To do so, we examined the ways in which family and social‐level factors were associated with changes in child mental health. We report the initial results concerning the effects of the first UK lockdown period on child and family well‐being and coping. We particularly focus on financial circumstances and on child and parental mental health.

## METHOD

### Sample

The participants were 142 children (aged 4–8 at initial assessment; mean age = 6 years and 2 months, and aged 5–10 at the time of assessment during lockdown; mean = 7 years and 8 months; 32% girls) previously identified by teachers or Special Educational Needs Co‐ordinators (SENCOs) as having emerging mental health problems at school.^3^ Emotional and behavioural problems exhibited in the classroom were assessed by the Strengths and Difficulties Questionnaire (SDQ) (R. Goodman, 1997). The children came from 67 mainstream schools in Wales. Children and families had been assessed pre‐COVID using detailed and well‐established face‐to‐face multi‐informant interview and questionnaire assessments of child mental health, social and family risk and protective factors, and task‐based assessments of cognitive and socio‐emotional functioning. Measures used in the present study included parent‐completed mental health measures relating to the child (e.g., the SDQ, [Goodman, 1997]; an anxiety measure [Screen for Child Anxiety Related Emotional Disorders, SCARED] [Birmaher et al., 1999], and to socio‐economic status. Children were excluded from the study if they had received a clinical diagnosis at the time of assessment. Children and families were reassessed during lockdown and school closure (between July 2020 and September 2020). The mean time between the first (pre‐COVID) and second (during pandemic and first lockdown) assessments was 17 months (range = 4–35 months), with pre‐COVID assessments taking place between September 2017 and March 2020. All procedures were ethically approved by Cardiff University (EC.20.06.09.6053RA).

### Procedure

At baseline (pre‐COVID), parents provided child and family background information, including details of household income, parental education, ethnic background and events indicating childhood adversity (see Table 1). Child adversity prevalence was denoted by parent's reports of physical abuse, parental separation, parental mental health problems and parental incarceration. Parents also reported on involvement of social services and Child and Adolescent Mental Health Services (CAMHS) and any extra support provided by the child's school for special educational needs (SEN). Measures of child mental health (SDQ, SCARED) and parental anxiety and depression (Hospital Anxiety and Depression Scale [HADS]) were also completed by parents.

**TABLE 1:** Participant demographics at baseline (Pre‐COVID) (*n* = 142)

	Percentage
Socio‐economic indicators	
WIMD quintiles (two most deprived categories)	49
Income (less than £20,000 pa)	37
Families including a keyworker	48
Parental education	
No formal educational qualification	11
O‐levels/General Certificate of Secondary Education (GCSEs)	33
A‐levels/Higher	24
University degree	17
Higher or postgraduate degree	16
Ethnicity	
White British	83
Other European	1
African	1
Asian	2
British/European	4
British/Asian	2
British/Caribbean	1
British/Turkish	1
Other	5
Child adversity	
Physical abuse present	49
Parental separation	23
Parental mental health problems	43
Parental incarceration	2
Child adversity sum (≥1)	66
Support	
Social services involvement	28
CAMHS involvement	8
Extra school support for SEN	60
Teacher‐reported SDQ*	
SDQ total, mean (SD)	17.17 (6.27)
% high/very high	64
SDQ internalising, mean (SD)	5.96 (3.51)
% high/very high	16
SDQ externalising, mean (SD)	11.18 (4.32)
% high/very high	53

Abbreviations: SDQ, Strengths and Difficulties Questionnaire; SEN, special educational needs; WIMD, Welsh Index of Multiple Deprivation.

*Significant correlations between teacher‐reported and parent‐reported internalising difficulties (*r* = 0.185, *p* < 0.05) and externalising difficulties (*r* = 0.275, *p* < 0.001) at Time 1 (time of referral). Higher parent–teacher agreement for externalising compared to internalising difficulties is commonly reported (Cheng et al., 2018).

We received funding early in July 2020 and re‐contacted families and children between July and early September (i.e., during first lockdown and school closure). Unless parents indicated significant time constraints due to work commitments or ongoing assessment with other services (e.g., CAMHS), families participated in researcher‐led video calls (mean interview duration 1.5 h) and repeated the measures of child mental health difficulties (parent SDQ, SCARED), and parental anxiety and depression (HADS). We also assessed COVID‐19 exposure (risk) and current status; key worker status; school provision and contact; change in employment status and financial stress; daily routine and lifestyle and time spent educating children and engaging with social contacts.

### Measures

#### Child mental health

The SDQ is a 25‐item screening questionnaire for mental health difficulties in children and young people aged 3–16 years. Parents rated their child's behaviour when the child was first assessed (pre‐COVID) and again during lockdown. The questionnaire consists of five subscales (emotional symptoms, hyperactivity/inattention, conduct problems, peer problems and prosocial behaviour). A total difficulties score (SDQ total) comprising the first four subscale scores indicates the overall extent of a child's mental health problems. Good discriminative validity has been reported in typical and high‐risk children (Mullick & Goodman, 2001) and the SDQ has been shown to be effective in screening for psychiatric disorders in community samples (Goodman et al., 2001). Additionally, a broader internalising subscale (combination of emotional and peer problems) and an externalising subscale (combination of conduct problems and hyperactivity) were created (Goodman et al., 2010). We categorised these broader subscales according to their recommended cut‐off points (Goodman et al., 2010) indicating a high/very high score (17 out of 40 for total difficulties, 9 out of 20 for the internalising scale and 12 out of 20 for the externalising scale). In the case of missing scores, scale means were calculated from the remaining valid items. SDQ items showed acceptable internal consistency for the internalising subscale (ranging from 0.66 to 0.67), externalising subscale (ranging from 0.69 to 0.74). Table 2 shows the percentage of children in each category. Teacher SDQ ratings of the children at time of referral are also reported (see Table 1).

**TABLE 2:** Mean scores for child mental health and parent mental health

	Time 1	Time 2
	Pre‐COVID	1st Lockdown
Parent‐reported SDQ		
SDQ total, mean (SD)	19.14* (6.43)	20.76* (6.45)
% high/very high	61%	69%
SDQ internalising, mean (SD)	6.86* (3.76)	8.18* (3.92)
% high/very high	34%	45%
Emotional	3.53* (2.49)	4.18* (2.48)
Peer	3.31* (2.18)	3.98* (2.52)
SDQ externalising, mean (SD)	12.25 (4.17)	12.54 (4.16)
% high/very high	58%	59%
Conduct	4.40 (2.69)	4.36 (2.63)
Hyperactivity/inattention	7.84 (2.34)	8.18 (2.22)
SCARED		
Total anxiety, mean (SD)	18.56* (13.67)	24.87* (14.37)
Panic/somatic symptoms	2.86** (3.75)	8.13** (5.33)
Generalised anxiety disorder	4.83* (4.20)	6.10* (3.66)
Separation anxiety	5.11* (3.94)	4.33* (3.04)
Social anxiety	4.83* (4.03)	3.80* (2.85)
School anxiety	1.21** (1.40)	2.59** (1.97)
HADS		
Total symptoms (mean)		15.75 (8.24)
Anxiety		
% Borderline level		27%
% Abnormal level		30%
Depression		
% Borderline level		26%
% Abnormal level		18%

Abbreviations: HADS, Hospital Anxiety and Depression Scale; SCARED, Screen for Child Anxiety Related Emotional Disorders; SDQ, Strengths and Difficulties Questionnaire.

*Difference between Time 1 and Time 2 significant at *p* < 0.05, **Difference between Time 1 and Time 2 significant at *p* < 0.001.

[Corrections made on 12 April 2021, after first online publication: This is now Table 2 in this version.]

The SCARED (Birmaher et al., 1999) was originally developed as a clinical screening tool for childhood anxiety disorders in clinical samples; however, research has also found it to be a valuable screening tool for anxiety disorders in community samples (Hale et al., 2005). The SCARED consists of 41 questions; responses are recorded by the parent as describing the child's behaviour during the previous 3 months. In the case of missing scores, scale means were calculated from the remaining valid items. A total score of ≥25 suggests the presence of an anxiety disorder. Subscales assess five specific anxiety disorders: panic/somatic anxiety, general anxiety, separation anxiety disorder, social phobia and school phobia. Items for each subscale of the SCARED measure revealed good internal consistency (ranging from 0.60 to 0.81).

#### Parental mental health: Anxiety and depression

The HADS (Zigmond & Snaith, 1983) is a 14‐item screening measure designed to assess symptoms of anxiety and depression in non‐psychiatric populations, identifying individuals at elevated risk for anxiety and depressive disorders. Scores range from 0 to 21, with scores from 8 to 10 indicating borderline levels and scores from 11 to 21 indicating abnormal levels warranting clinical assessment. In the case of missing scores, scale means were calculated from the remaining valid items. This measure has been shown to have sensitivities of 82% and 70%, and specificities of 94% and 68%, for depressive and anxiety disorders, respectively (Barczak et al., 1988). HADS scores also revealed good internal consistency (ranging from 0.73 to 0.83).

#### Financial stress

A dichotomous variable was computed to reflect whether families had experienced financial stress (‘0’ for families experiencing no financial stress and ‘1’ for families reporting one or more indicators of financial stress) for use in subsequent correlation and regression analyses. Families were classified as experiencing financial stress when parents reported during the video call that they had lost employment, lost a significant amount of their income, struggled to pay bills, were at risk of eviction or losing their accommodation, were unable to afford to buy sufficient food, or had had to use emergency loans or foodbanks.

## DATA ANALYSIS

Data analyses were conducted using SPSS (IBM Corp, 2019). Prior to the statistical analyses, the distributions of the key measures (i.e., SDQ, SCARED, HADS) were examined. Scores for all measures were found to be normally distributed. Next, descriptive statistics for the sample were calculated. Child and family characteristics (including prevalence variables [i.e., change in SDQ classification from Time 1 to Time 2], financial stress and categories of parental mental health difficulties for anxiety and depression) are shown as *n* (%). Continuous variables for measures of child and parent mental health are described as the mean ± standard deviation (SD). Changes in continuous variables for measures of child and parent mental health from Time 1 to Time 2 were then analysed using independent sample *t*‐tests and measures of effect size are reported.

Correlations between the variables of interest were conducted prior to the main analyses. Mediation analyses were performed to assess whether parental mental health mediated the relationship between financial stress and total child difficulties at Time 2 (controlling for total difficulties at Time 1 and the duration between Time 1 and Time 2 assessments). The total and direct and indirect effects, reflecting the unstandardised regression coefficients between the predictors, mediators and outcome variables in each model, were computed using the PROCESS macro (Hayes, 2013) for SPSS, with bootstrapping used to test the significance of all the effects. A resampling procedure of 5000 bootstrap samples was applied, providing estimates and 95% confidence intervals (CIs) for the indirect effects. The indirect effects were considered significant when the CI did not include zero.

## RESULTS

Descriptive statistics are shown in Table 1. There it can be seen that nearly half of the sample of 142 children fell into the two highest categories of the Welsh Index of Multiple Deprivation (WIMD; Welsh Assembly Government, 2019). Over one‐third of the sample had a household income of less than £20,000 per annum. These families were considered to be living in poverty, based on the UK household income poverty definition (https://commonslibrary.parliament.uk/research‐briefings/sn07096/), which uses a household income <£20,000 to indicate poverty. A large proportion of the parents (44%) had no post‐16 educational qualifications. The vast majority of the families identified as white British. Nearly three‐quarters of the children had one or more adverse child experience, and just under one‐third of the families reported that they had been involved with social services. Teachers’ ratings on the SDQ (Goodman, 1997) showed that 64% of these children were at high or very high risk of mental health problems when they were initially referred (see Table 1). Together, these data show that there was substantial deprivation and child mental health difficulties in this sample.

### Change in financial circumstances, child mental health and parental mental health

Eighty‐one families (57%) were living in poverty at Time 1 or reported to have experienced significant financial difficulties during the first lockdown. Table 3 shows the extent to which families in our sample experienced financial stress during lockdown. It is evident that a higher proportion of low income families experienced some degree of financial setback, with 41% reporting one or more indicators of financial stress compared to 33% of higher income families.

**TABLE 3:** Summary of financial difficulties experienced during the first lockdown by income group

	%
High‐income families (>£20,000)	
% Reporting >1 indicator of financial stress	33
Low‐income families (<£20,000)	
% Reporting >1 indicator of financial stress	41

*Note:* Financial stress: losing employment, losing significant amount of income, struggling to pay bills, being at risk of eviction or losing accommodation, unable to afford sufficient food, having to use emergency loans or foodbanks.

[Corrections made on 12 April 2021, after first online publication: This is now Table 3 in this version and the ‘%’ values have been corrected.]

Table 2 shows mean scores and descriptive statistics for our key measures of child mental health and parental mental health, along with comparisons between Time 1 (pre‐COVID) and Time 2 (first lockdown) scores, where both sets of measures are available. There was a significant increase in child mental health problems, with a significant change in total SDQ difficulties from pre‐COVID levels (*t*(139) = −3.39, *p* < 0.05, *d* = −0.29). It is evident that this was primarily attributable to an increase in internalising problems (*t*(139) = −4.02, *p* < 0.001, *d* = −0.34). Despite a small increase in hyperactivity/inattention difficulties, no significant change was found for externalising problems (*t*(140) = −1.13, *p* = 0.261). Our clinical measure of child anxiety (SCARED) revealed significant increases in total scores (*t*(121) = −5.42, *p* < 0.001, *d* = −0.49), with significant increases in all subscales apart from a significant reduction in separation anxiety and social anxiety. Table 2 only presents parent mental health (HADS) at Time 2 because there was a lower completion rate (*n* = 79) for the HADS pre‐COVID, due to time restrictions. However, there were no significant differences on any key variables between parents with and without pre‐COVID HADS data. However, it is worth noting that for parents with both Time 1 and Time 2 HADS data, there was a significant increase in total HADS scores from Time 1 (*M* = 11.81, *SD* = 5.23) to Time 2 (*M* = 14.79, *SD* = 7.88) (*t*(71) = −3.10, *p* < 0.05, *d* = 0.37). Furthermore, there was a significant increase in child mental health after controlling for parental mental health (SDQ total: *F*(1, 122) = 15.36, *p* < 0.001, *ηp2* = 0.112).

### Associations between financial stress, and parent and child mental health

Correlations were computed to assess the relations between financial stress, and child and parental mental health during lockdown. The resulting correlation coefficients are shown in Table 4. It can be seen that there was a strong relation between Time 1 and Time 2 SDQ scores. Another finding worth noting is that family financial stress was significantly correlated with parental mental health, and there was a significant correlation between parental mental health and child mental health as indexed by SDQ and HADS scores.

**TABLE 4:** Correlations between measures of parent (HADS) and child (SDQ) mental health and financial stress during first lockdown

	1	2	3	4
1. SDQ total (Time 1)				
2. SDQ total (Time 2)	0.618**			
3. Parental mental health (HADS total)	0.086	0.340**		
4. Financial stress	−0.054	0.138	0.246**	

Abbreviations: HADS, Hospital Anxiety and Depression Scale; SDQ, Strengths and Difficulties Questionnaire.

**Significant at *p* < 0.001.

Next, we used mediation analysis to assess the relationship between financial stress, parental mental health (HADS total score) and child mental health (SDQ total score) during the lockdown (Time 2). We examined whether parental mental health mediated the relationship between financial stress and child mental health during lockdown, controlling for pre‐COVID SDQ total scores (Time 1) and the length of time between the Time 1 and Time 2 assessments.

**FIGURE 1:**
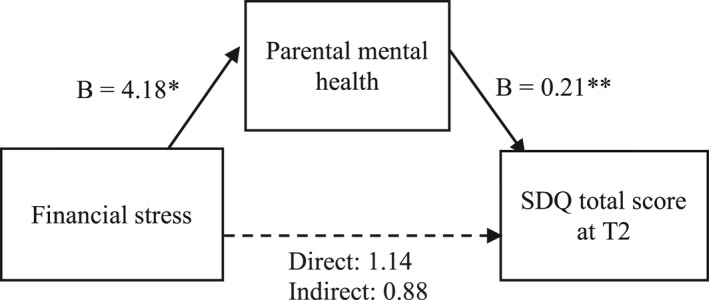
Results of mediation analysis predicting Strengths and Difficulties Questionnaire (SDQ) total score at Time 2, controlling for SDQ score at Time 1 and length of time between assessments, from financial stress and parental health during first lockdown. **p < 0.01

The results of the analysis, summarised in Figure 1, show that the total effect model explains 39% of the variance in the SDQ score. Financial stress was significantly related to parental mental health, *B* = 4.18, SE = 1.51, *p* < 0.05. Parental mental health was significantly related to SDQ score, *B* = 0.21, SE = 0.05, *p* < 0.001. The direct effect of financial stress on SDQ score was not significant, *B* = 1.14, SE = 0.93, *p* = 0.224. The mediating role of parental mental health was assessed by the indirect effect of financial stress on SDQ total score, via parental mental health. The value of this effect was 0.88, 95% CI (0.17, 1.88). Because the CI does not include zero, we conclude that the indirect effect is significant.

## DISCUSSION

The results show that the lockdown resulting from COVID‐19 had a significantly negative impact on financial security, child mental health and parental mental health. We found high levels of mental health problems in children during lockdown, with 69% of the sample having high or very high SDQ total scores, significantly higher than the already high‐level pre‐COVID. There was, in particular, a significant increase in internalising problems, rising from 34% to 45% in the sample. When we examined the specific types of anxiety that the children experienced, our clinical measure of child anxiety (SCARED) revealed significant increases in generalised anxiety, panic and somatic symptoms, and school anxiety, whereas there was a reduction in social and separation anxiety. It seems likely that exposure of children to parental and media discussions about death/dying, illness, risks and the need to take precautionary measures made children more aware of and more self‐focused on bodily sensations, more worried about dying, perhaps even giving rise to panic. At the same time, worries about schoolwork or home‐schooling, not being at school or going back to school, are likely reasons for the increase in school anxiety. Our in‐depth qualitative interview data with parent and child should help to clarify some of these findings.

It is interesting that we did not find an increase in externalising problems, given that this sample had high levels of externalising problems to begin with, as evidenced in ratings by parents and teachers, at the time of referral. Children with hyperactivity and conduct problems often find school stressful, and it seems possible that some children improved with respect to their externalising problems because they were calmer without the stress of having to go to school.

During lockdown, parents reported high levels of anxiety (27% having borderline scores, and a further 30% abnormal scores) and depression (26% borderline, 18% abnormal). Our results show that both the deterioration in child mental health and the high prevalence of parental mental health problems were significantly related to family financial stress. While 37% of the sample was already living in poverty pre‐lockdown, 22% reported significant loss of income during lockdown. Probably as a result, 14% of families reported struggling to pay bills and 9% struggled to afford food. Further, a higher proportion of families living in poverty pre‐lockdown reported experiencing financial stress during the lockdown, demonstrating the adverse economic effects of the lockdown on already poor families. Poverty leads to a range of adversities, including malnutrition but also inconsistent caregiving, neglect or maltreatment, all of which can lead to high levels of sustained and uncontrollable stress (Blair & Raver, 2016). Inconsistent schedules and lack of family routines make it difficult for children to predict and anticipate sequences of events, and these lifestyle factors elicit high levels of chronic and recurring stress—effects that are likely to increase anxiety and affect behavioural problems (Shonkoff et al., 2012). The mental health gap between advantaged and disadvantaged children has not reduced over the last 20 years (Collishaw et al., 2019); a major concern is that this gap may now increase.

Although our mediation analysis is based on correlational data and therefore open to the critique that no firm conclusions can be drawn regarding causality, we found evidence consistent with the view that financial strain has an adverse effect on parental mental health, which in turn adversely affects child mental health (Yoshikawa et al., 2012). Importantly, however, the interpretation of our mediation model needs to take into account the use of a single informant (i.e., parents) for all measures included in the analysis. Research has pointed to the role of the informant's psychological functioning in rating of children's behaviour (Treutler & Epkins, 2003; Youngstrom et al., 1999). As a consequence of the lockdown and school closures, and therefore a lack of contact with the children, it was simply not possible for teachers and SENCOs to complete the SDQ at Time 2. It is also worth noting that there were high levels of agreement between teacher and parent ratings of child mental health difficulties (SDQ) pre‐COVID, for both internalising and externalising problems, and that the significant increase in child mental health from Time 1 to Time 2 remained after controlling for parental mental health.

Overall, the results provide suggestive evidence regarding how best to intervene in a way that would improve children’s mental health. Most obviously, families need financial support, but steps to support parents’ mental health should also help to protect children’s mental health.

The fact that there was heterogeneity in our vulnerable sample with respect to adaptation and mental health is also worth noting. Children who fared better during lockdown had parents who reported less or no financial insecurity and fewer anxiety or depression symptoms. Carefully assessing the child and family factors that are most strongly associated with better or worse outcomes should help to inform policies designed to enhance children’s resilience during and after COVID‐19; it should also inform the design of novel child‐ and family‐focused interventions intended to promote child resilience.

The children in our study had a range of difficulties prior to lockdown. Our clinical screening showed that teachers rated 64% of the sample as being at high or very high risk of mental health problems before lockdown. Whilst teachers/SENCOs referred children mainly because they were perceived as disruptive (53%), emotional problems were still a major problem (34% of sample, despite only 16% being referred for emotional difficulties). Longitudinal studies using the SDQ in young children have shown that externalising symptoms, especially hyperactivity problems (relative to other symptom domains) are most predictive of mental health problems (including hyperkinetic, behavioural and emotional disorders) in adolescence (Nielsen et al., 2019). Emotional disorders have a later onset than externalising behaviours, which helps to explain the lower proportion of emotional problems in our children before the pandemic; however, the significant increase in emotional problems in these children during lockdown (from 34% to 45%) is a cause for concern.

To our knowledge, this is the first study to highlight the severe impact of COVID‐19 on already vulnerable children and families. The findings make distressing reading, especially when seen in the context of continuing economic uncertainty and the likelihood of higher unemployment. Food insecurity is amongst the strongest predictors of mental health outcomes (Cluver et al., 2020; Collishaw, Hammerton, et al., 2016). Our analyses show how the financial stress created by the pandemic is associated with, and possibly responsible for, increased mental health problems in children through its impact on parental mental health.

## CONFLICTS OF INTEREST

No conflicts declared.

## AUTHOR CONTRIBUTIONS

Dolapo Adegboye: data curation; formal analysis; investigation; methodology; software; supervision; validation; visualisation; writing – original draft; writing ‐ review and editing. Ffion Williams: data curation; formal analysis; investigation; methodology; software; visualisation. Stephan Collishaw: conceptualisation; funding acquisition; methodology; writing – original draft; writing ‐ review and editing. Katherine Shelton: conceptualisation; funding acquisition; methodology; writing – original draft; writing ‐ review and editing. Kate Langley: conceptualisation; funding acquisition; methodology; writing – original draft; writing ‐ review and editing. Christopher Hobson: conceptualisation; funding acquisition; methodology; writing – original draft; writing ‐ review and editing. Daniel Burley: curation; investigation; methodology; software. Stephanie van Goozen: conceptualisation; formal analysis; funding acquisition; methodology; project administration; supervision; validation; visualisation; writing – original draft; writing ‐ review and editing.

## Data Availability

The data that support the findings of this study are available from the corresponding author upon reasonable request.

## ENDNOTES

^1^

https://www.who.int/emergencies/diseases/novel‐coronavirus‐2019/advice‐for‐public/healthy‐parenting

^2^

https://cpag.org.uk/policy‐and‐campaigns/report/poverty‐pandemic‐impact‐coronavirus‐low‐income‐families‐and‐children

^3^

http://www.cardiff.ac.uk/research/explore/research‐units/neurodevelopment‐assessment‐unit
